# Predictive factors for degenerative lumbar spinal stenosis: a model obtained from a machine learning algorithm technique

**DOI:** 10.1186/s12891-023-06330-z

**Published:** 2023-03-23

**Authors:** Janan Abbas, Malik Yousef, Natan Peled, Israel Hershkovitz, Kamal Hamoud

**Affiliations:** 1 Department of Physical Therapy, Zefat Academic College, 13206 Zefat, Israel; 2 Department of Anatomy and Anthropology, Sackler Faculty of Medicine, Tel Aviv University, 6997801 Tel Aviv, Israel; 3 Department of Information Systems, Zefat Academic College, Zefat, Israel; 4 Department of Radiology, Carmel Medical Center, 3436212 Haifa, Israel

**Keywords:** Degenerative lumbar spinal stenosis, Machine learning, Computer Tomography, Spine dimensions

## Abstract

**Background:**

Degenerative lumbar spinal stenosis (DLSS) is the most common spine disease in the elderly population. It is usually associated with lumbar spine joints/or ligaments degeneration. Machine learning technique is an exclusive method for handling big data analysis; however, the development of this method for spine pathology is rare. This study aims to detect the essential variables that predict the development of symptomatic DLSS using the random forest of machine learning (ML) algorithms technique.

**Methods:**

A retrospective study with two groups of individuals. The first included 165 with symptomatic DLSS (sex ratio 80 M/85F), and the second included 180 individuals from the general population (sex ratio: 90 M/90F) without lumbar spinal stenosis symptoms. Lumbar spine measurements such as vertebral or spinal canal diameters from L1 to S1 were conducted on computerized tomography (CT) images. Demographic and health data of all the participants (e.g., body mass index and diabetes mellitus) were also recorded.

**Results:**

The decision tree model of ML demonstrate that the anteroposterior diameter of the bony canal at L5 (males) and L4 (females) levels have the greatest stimulus for symptomatic DLSS (scores of 1 and 0.938). In addition, combination of these variables with other lumbar spine features is mandatory for developing the DLSS.

**Conclusions:**

Our results indicate that combination of lumbar spine characteristics such as bony canal and vertebral body dimensions rather than the presence of a sole variable is highly associated with symptomatic DLSS onset.

## Introduction

Degenerative lumbar spinal stenosis (DLSS) is the most common spine disease in the elderly population [1]. Moreover, this pathology is most closely correlated with lumbar spine surgery performed on this population [2]. It is well accepted that the radiological manifestations of DLSS are degeneration of the three-joint complex, ligamentum flavum thickening and osteophytes formation [3, 4], which ultimately decrease the space available for the neurovascular elements. However, the essential definition of DLSS combines the radiological picture with the presence of clinical criteria following the Spine Patient Outcomes Research Trial (SPORT) [5]. It is noteworthy that up to 30% of older individuals (≥ 55 years) have at least moderate radiological stenosis without symptoms [6].

Many studies have previously reported that there are anthropometric elements as well as morphological spine characterizations that significantly associate with symptomatic DLSS. For example, greater BMI, vertebral body size and lesser anterior posterior bony canal diameters increase the risk for DLSS development [7, 8]. On the other hand, the precise pathogenesis of this phenomenon is still inconsistent and unclear. As a poor correlation between the clinical picture and the radiological signs has been observed, there is a vital need to identity those individuals who will later suffer from symptomatic DLSS.

Machine learning (ML) is defined as a series of computational tools that are capable of determining the association between material data and do not required a specific setup [9]. In general, ML is applied in fields requiring predictions or decisions to be made according to the training data. ML has three major subgroups: supervised learning, unsupervised learning and reinforcement learning [10, 11]. Supervised ML is the subgroup in which a learner describes the input–output relationship based on labeled input variables with a grounded truth [12]. It is also a model used to analyze the training data to synthesize the pattern between independent and dependent variables [13], then the testing dataset can to be predicted. One of the three common ML models is the decision tree (DT) learning whose classification and regression implements a grouping or a regression task, which is more visible and easier to understand than other modalities [13]. The tree comprises internal nodes (conditions), branches (decisions) and leaves (end) that are not computationally intensive and therefore suitable for big data [14, 15].

To the best of our knowledge, no previous study has dealt with big data and has examined all the variables that together could predict DLSS development in order to highlight the most associated one. In addition, using a specific and exclusive method such as ML is rare in this field. In general, there is a belief that ML may provide new insights into biomedical analyses and diseases [16].

In recent years, the potential role of ML in driving personalized medicine has become highly recognized, especially in the realm of spine care [17–20]. Moreover, ML models are common in rheumatology, where numerous classification algorithms have been developed [21–24].

Compared with conventional associated factors obtained by the logistic regression analysis, ML has numerous advantages [18]. Firstly, fewer restrictions exist on the number of variables or predictors used in the final model with ML, thus making large modern datasets more amenable to ML approach [25]. Secondly, it can capture non-linear relationships between the predictor variables and the outcome variable, which it can exhibit complex interactions and relationships that may not be captured by traditional statistical models such as logistic regression. Thirdly, ML is less sensitive to outliers than logistic regression, because it uses a combination of multiple decision trees, so the effect of outliers is dampened. Finally, the ability of this method to handle missing and big data, contrary to traditional statistical analysis, will considerably improve diagnostic accuracy and prognosis [26].

The aim of this study is to reveal the outstanding predictive variables for the development of symptomatic DLSS using the ML algorithm technique.

## Materials and methods

### Study design and participants

This is a retrospective study that includes two groups: individuals with DLSS (*n* = 165) and control (*n* = 180). The study groups were enrolled between 2008 and 2012 and included demographic (e.g., age and occupation) and health data (hypertension, diabetes mellitus) regarding the participants [7]. Details about the inclusion and exclusion criteria for the study groups could be obtained from the research of Abbas and colleagues [8] as it is the same sample of participants. One of the coauthor (KH) who is a spine surgeon has recruited the participants of the DLSS group following the SPORT recommendations [5]. The predominant symptom of individuals with DLSS was neurogenic claudication that usually improves in sitting or lumbar flexion and worsens with standing and lumbar extension. All participants underwent computer tomography (CT) (Brilliance 64, Philips Medical System, Cleveland, OH, thickness of the sections were 1–3 mm and MAS, 80–250) in the supine position with extended knees.

All the CT measurements were taken from L1 to S1 levels and included the vertebral body diameters (width, length and height), bony canal dimensions such as anterior- posterior (AP), medio-lateral, and cross-section area (CSA) [8]. We also addressed the facets orientation and tropism [27], pedicle width and height [28], spinous process orientation [29], laminar inclination and inter-laminar angle [8]. Spine pathology such as vacuum phenomenon, intervertebral disc height, and the presence of Schmorl’s nodes [30, 31] were also recorded. Dimensions of the para-vertebral muscles (psoas, multifidus and erector spinae) density and CSA [32] as well as the spino-transvers area were evaluated in the axial plane at the middle part of L3 vertebra. The presence of lumbosacral transitional vertebra, sacral slope and lumbar lordosis angles were also recorded [33]. It should be noted, that cases with LF hypertrophy, facet joints arthrosis, degenerative spondylolisthesis and intervertebral disc bulging, which are the main radiological manifestations of DLSS, were enrolled in our study, however, these variables, were not considered in the machine learning analysis. All the participants gave informed consent to participate in this study. The Departmental Research Ethics Committee, of the Carmel Medical Center (0083–07-CMC), approved this study.

### Statistical analyses

We used SPSS version 20, in order to check the normal distribution for all the metric parameters. Descriptive statistics (frequencies and number) were also used to present the distribution of age, BMI and CSAs of dural sac of the participants in the study groups.

### Supervised machine learning analysis

**Fig. 1:**
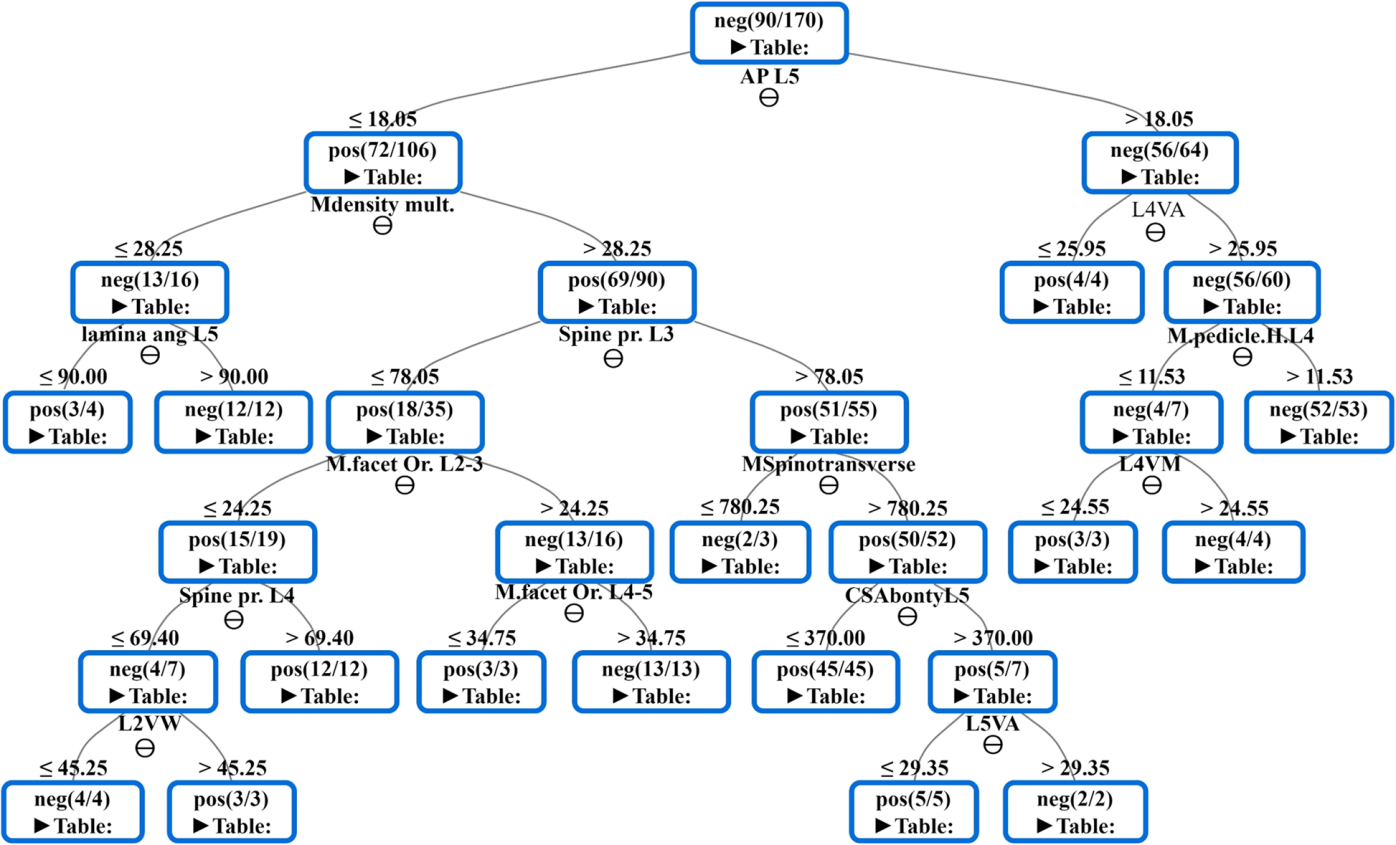
The decision tree (DT) of random forest obtained by the given variables in males. "AP- antero-posterior bony canal, Mdensity mult- mean density of multifidus, VA- vertebral height anterior, lamina ang—inter-laminar angle, Spine pr.—spine process inclination, M.facet Or.- mean facet orientation, Mspinotransverse- mean spinotransverse area, CSAbony- cross section area of bony canal VM- vertebral height middle, M.pedicle.H- mean pedicle height, VW- vertebral body width"

**Fig. 2:**
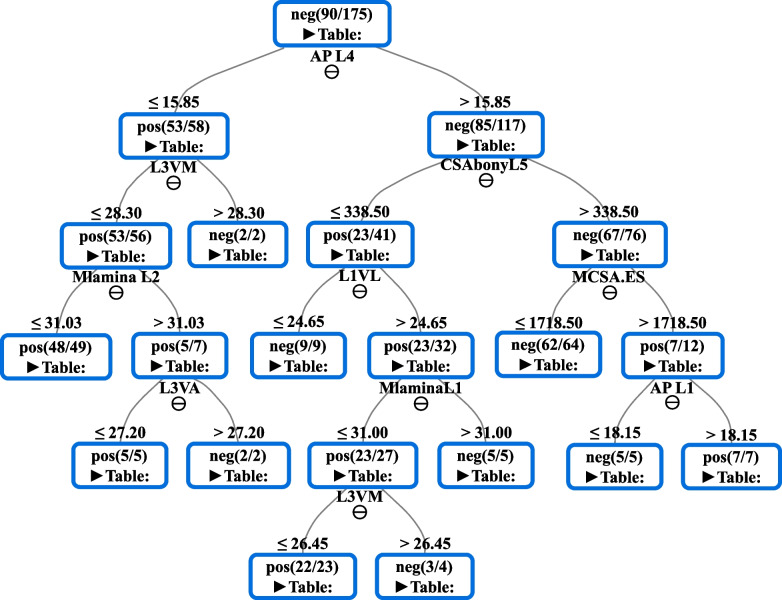
The decision tree (DT) of random forest obtained by the given variables in females. "AP- anterio-posterior bony canal, VM- vertebral height middle, CSAbony- cross section area of bony canal, Mlamina- mean lamina inclination, VL- vertebral body length, MCSA.ES- mean cross section area of erector spina muscle, VA- vertebral height anterior"

We have applied the supervised machine learning approach considering the random forest (RF) for the classification task [34] in view of the default parameters (Split criteria: Information Gain Ratio and number of trees 100). The RF also provides a score that expresses the significance of each variable or feature. Significant variables are essential for developing the final model, whereas those with a low score could be removed from the final model. For simple visualization, we have used the decision tree (DT) model with the default parameters (Quality measure is Gini index, Pruning method is Minimum Descriptive Length). DT can be expressed as a set of "if–then-else" decision rules. The DT is a tree consisting of root nodes and leaf nodes. A decision node has two or more branches. A leaf node represents a classification or decision [35] ("pos" or "neg" label) (Figs. 1 and 2). The peak of the DT corresponds to the best predictor variable called the root node. For using the DT as a classifier, one has to start from the root node, moving to the next node (branches) based on the decision rules. This process is repeated until reaching the leaf node (ends) that explains the prediction outcome ("pos" = DLSS or "neg" = Control) (Figs. 1 and 2). The RF classifier was trained and tested with a split into 90% training and 10% testing data. The features of the RF model were recorded over all the 100 iterations. The average scores were calculated to assign a final score to each feature. The higher score outcomes indicate a significant impact of the feature to the model.

We evaluated the performance of RF classifier by the following measures: (1) sensitivity (SE) which represent the true positive (TP) rate, (2) specificity (SP) which represents the true negative (TN) rate (complement of sensitivity), and (3) precision (PR) which represents the ability to correctly predict the positive target condition to the total [28]. In addition, we assessed the accuracy (ACC) which represents the classifier ability to predict the target condition correctly, and the F-measure that illustrates the classifier ability to predict the target condition correctly (compared to ACC, it is more accurate in cases of imbalanced data set, since it considers both PR and SE) [35]. All reported performance measures refer to the average of 100-fold Monte Carlo cross validation (MCCV) [36].

$$\mathrm{Sensitivity}=\mathrm{TP}/(\mathrm{TP}+\mathrm{FN}),\;\mathrm{Specificity}=\mathrm{TN}/(\mathrm{TN}+\mathrm{FP}),\;\mathrm{Precision}=\mathrm{TP}/(\mathrm{TP}+\mathrm{FP}),\;\mathrm{Accuracy}=(\mathrm{TP}+\mathrm{TN})/(\mathrm{TP}+\mathrm{FN}+\mathrm{TN}+\mathrm{FP})$$

$$\mathrm{F}-\mathrm{ Measure }= 2\mathrm{ x }(\mathrm{PR x SE})/(\mathrm{PR }+\mathrm{SE})$$

## Results

Information concerning age, body mass index (BMI) and CSAs of dural sac of the studied groups is presented in Tables 1 and 2. No significant differences were found in the mean-age of the control group compared to the stenosis group: 62.8 ± 12 (males), 62 ± 12 (females) vs. 66.2 ± 11 and 62 ± 8, respectively (*P* > 0.05). Individuals in the stenosis group manifested higher values of BMI compared to their counterparts in the control group: 28.9 ± 4 (males), 31.4 ± 5 (females) vs. 27.3 ± 4, 27.6 ± 5, respectively (*P* < 0.05).

**Table 1:** Data concerning age and body mass index (BMI) in the studied groups by gender

Variables	Control (*n* = 180)		Stenosis (*n* = 165)	
	Males (*n* = 90)	Females (*n* = 90)	Males (*n* = 80)	Females (*n* = 85)
Age (%):				
40–60 years	48 (*n* = 43)	49 (*n* = 44)	28 (*n* = 22)	41 (*n* = 35)
61–90 years	52 (*n* = 47)	50 (*n* = 45)	72 (*n* = 58)	59 (50)
> 90 years	0	1 (*n* = 1)	0	0
BMI (%):				
18.5—24.9	27 (*n* = 24)	33 (*n* = 30)	19 (*n* = 15)	11 (*n* = 9)
25- 29.9	50 (*n* = 45)	35 (*n* = 31)	41 (*n* = 33)	33 (*n* = 28)
≥ 30	23 (*n* = 21)	32 (*n* = 28)	40 (*n* = 32)	56 (48)

**Table 2:** Percentage of subjects with cross section area (CSA) of dural sac below 70 mm^2^, between 70–99.9 mm^2^ and ≥ 100 mm^2^ in the studied groups by level

Levels	Control group (*n* = 180)			Stenosis group (*n* = 165)		
	Below 70 mm^2^	70–99.9 mm^2^	≥ 100 mm^2^	Below 70 mm^2^	70–99.9 mm^2^	≥ 100 mm^2^
L1	0	0	100 (*n* = 180)	7 (*n* = 11)	9 (*n* = 15)	84 (*n* = 139)
L2	0	3 (*n* = 5)	97 (*n* = 175)	27 (*n* = 44)	23 (*n* = 38)	50 (*n* = 83)
L3	3 (*n* = 5)	9 (*n* = 16)	88 (*n* = 159)	61 (*n* = 101)	26 (*n* = 43)	13 (*n* = 21)
L4	4 (*n* = 7)	14 (*n* = 25)	82 (*n* = 148)	87 (*n* = 144)	8 (*n* = 13)	5 (*n* = 8)
L5	3 (*n* = 5)	14 (*n* = 25)	83 (*n* = 150)	32 (*n* = 53)	27 (*n* = 44)	41 (*n* = 68)

The results showed that 50% to 72% of the participants in both groups are between the ages of 61and 90 years. We also found that almost half of the stenosis group (48%) suffered from obesity (BMI ≥ 30) compared to 28% in the control group. It is significant that an average of 74% of the stenosis group at L3 and L4 levels manifested CSAs of the dural sac below 70 mm^2^ compared to 4% in the control group.

Due to gender dimorphism as well as hormonal or lifestyle differences, we carried out the ML analysis for each gender separately. The DTs demonstrate the significant variables/features that predict the development of DLSS in both genders independently (Figs. 1 and 2). The results reveal 14 features for males as opposed to nine features in the female group (Figs. 1 and 2). The score averages of each variable that is involved in the DTs are presented in Table 3. The score is an indication of the importance of the variable in the model. The value of 0 means the variable is irrelevant, while value of 1 means that is mostly relevant.

**Table 3:** Scores of the features that were involved in the DTs for males and females independently

Gender	Variable	Score
Male	AP L5	1
	L4 Vertebral height anterior	0.496
	Mean density multifidus	0.325
	Inter-laminar angle L5	0.197
	Spinous process inclination L3	0.202
	Mean pedicle height L4	0.117
	Mean facet orientation L2-3	0.125
	Spino-transverse area	0.285
	L4 vertebral height middle	0.113
	Spinous process inclination L4	0.150
	Mean facet orientation L4-5	0.292
	CSA of bony canal L5	0.533
	L2 vertebral width	0.261
	L5 vertebral height anterior	0.398
Female	AP L4	0.938
	L3 vertebral height middle	0.095
	CSA of bony canal L5	0.674
	Mean lamina inclination L2	0.196
	L1 vertebral length	0.232
	CSA of erector spinae	0.242
	L3 Vertebral height anterior	0.192
	Mean lamina inclination L1	0.223
	AP L1	0.096

*AP* Anteroposterior, *CSA* Cross section area

We found that the AP diameter of the bony canal (L5), density of Multifidus, anterior and/or middle vertebral height (L4, L5), inter-laminar angle (L5), spinous process inclination (L3, L4), pedicle height (L4), facet orientation (L2-3, L4-5), spino-transverse area and vertebral body width (L2) are the best predictors for DLSS development in males (Fig. 1). In females, those were the AP diameter of the bony canal (L4, L1), anterior and/or middle vertebral height (L3), bony CSA (L5), laminar inclination (L1, L2), vertebral body length (L1), and CSA of erector spinae muscle (Fig. 2).

It should be noted that the AP bony diameters of L5 and L4 (peak of the trees) in males and females are the variables most significantly associated with DLSS. As we descend from the tree root (peak) to its leaf (edge), the impact of these variables /features decreases; therefore, one can conclude that the AP bony canal diameter of L5 in males is more significant than the facet orientation at L2-3 for DLSS onset (Fig. 1). We also considered the path from the root to the leaf as rules connected by an "and" relationship. In females, for example, when the "AP diameter at L4 is ≤ 15.58 mm and the middle vertebral body height of L3 is < 28.30 mm and the lamina inclination of L3 < 31.03", we have 48 subjects with DLSS from the total of 49. On the other hand, we have also observed stenotic females whose AP bony canal value of L4 is greater than 15.58 mm. This will occur with the following conditions: (a) the AP diameter of the bony canal of L4 > 15.58 mm, (b) bony canal CSA of L5 > 338.5 mm^2^, (c) CSA of erector spinae muscles > 1718.5 mm^2^ and (d) the AP diameter of the bony canal of L1 > 18.15 mm. We observed seven individuals in this situation with DLSS (*N* = 7) (Fig. 2). Additionally,the DT indicates that the impact of L4 AP bony canal diameter in females for developing DLSS is extraordinary when its values are ≤ 15.85 mm (left branches) rather than value of > 15.85 mm. The reason is that we have a greater number of "pos"/DLSS of leaf nodes on the left branches: 53 compared to 29 subjects with DLSS. This situation is also true in relation to the DT of males (Fig. 1).

## Discussion

To the best of our knowledge, this is the first study that presents a predictive model for symptomatic DLSS using machine-learning algorithms. Furthermore, it is the first study utilizing big data combining comprehensive parameters of the spine as well as health and demographic information.

The potential role of ML in recent years in driving personalized medicine has been well accepted. The use of ML in spine care and for lumbar degenerative disease is still in its infancy. Huber and colleagues [17], for example, have recently reported that texture analysis with ML offers highly reproducible quantitative parameters that increase accuracy for detecting severe lumbar spinal stenosis. ML algorithms indicate that fewer comorbidities with certain sociodemographic factors increased the likelihood of achieving minimal clinically important differences, which assist surgeons in determining the relevance and timing of surgery [37]. Others have also used ML to detect the preoperative predictive factors that could promote recovery and personalized shared decision-making [38, 39].

The outcomes of this study show that lumbar spine characteristics, rather than the demographic (e.g., age and BMI) and health data, are far more important factors that lead to development of symptomatic DLSS. Our results indicate that the combination of these spine features is mandatory for the development of this phenomenon. For example, the presence of a sole variable such as decreased value of bony canal or vertebral height diameter is not sufficient for DLSS development. Compared with conventional logistic regression analysis, the ML has superior advantages for revealing the most important predictive factors for DLSS development. Therefore, we think that the ML technique of analyzing the effect of different parameters is far more comprehensive and conclusive than utilizing the traditional statistical analysis using the odds ratio. The results of DTs show that the AP diameter of the bony canal at L5 and L4 levels for males and females respectively, has the greatest impact (scores of 1 and 0.938) upon DLSS onset for both genders. Bony spinal canal dimensions (e.g., AP diameter) are determined by genetics and/or environmental factors [40]. It should be noted that the AP diameter of the bony canal (L4 and L5) combined with other spinal features lead to DLSS development regardless of their values. However, the influence of the AP diameter is greater when its values fall in the low range. This result is in agreement with our previous study, which reported that the AP diameter of the bony canal has a significant role for development of DLSS [8]. We believe that this result may suggest that (a) the current view of the AP bony canal in DLSS pathology should be modified and (b) this variable should be considered an essential trigger for DLSS development.

The fact that DT peaks such as the AP diameter of the bony canal for each gender are at different levels (L5 vs. L4) could be explained by the study of Hay and colleagues (2015), which reported that the shape and curve of the lumbar lordosis are different between males and females [41].

Our results also indicate that both anterior and posterior elements of lumbar vertebra as well as the paravertebral muscles are involved in DLSS development. The vertebral body length, width and height belong to the anterior part, whereas the pedicle height, canal dimensions (AP and CSA), and facets inclination share the posterior portion. The para vertebral muscles have a crucial role in maintaining the stability of the spine segment [42]. There is a consensus that DLSS pathophysiology is based mainly on the spinal destructive and re-constructive changes [43]. We believe that lumbar spine variations at any part of the vertebra irrespective of its location, shared with the surrounding muscle could alter the forces trajectories upon the spinal column. These forces may harm the spine segment instability leading eventually to three-joint complex degeneration and stenosis.

### Study limitations

As this is a retrospective study, no causal relationship is determined. This study did not address in detail the pathogenesis of symptomatic DLSS. In addition, the CT scans were performed in supine position ignoring the effect of posture/or dynamic elements on radiological features. Further research with larger number of participants and data regarding their clinical presentation could be essential to improve the outcomes of ML methods in this field.

## Conclusions

Our study showed, using the decision tree method of machine learning, that lumbar bony AP canal is the strongest feature associated with DLSS. We also indicate that the combination of this feature with other variables obtained in the RF (e.g., paraspinal muscles morphology, vertebral body size) rather than any single variable is required for the onset of symptomatic DLSS. We believe that intervention programs or strategies that could affect the characteristics of the lumbar spine, such as the paraspinal muscles morphology and the vertebral body height, should be considered for the middle-age population in order to minimize the possibility of late onset of DLSS.

## Data Availability

The datasets generated and/or analyzed during the current study are not publicly available but are available from the corresponding author on reasonable request.

## Abbreviations

DLSS: Degenerative lumbar spinal stenosis
SPORT: Spine Patient Outcomes Research Trial
CSA: Cross-section area
ML: Machine learning
RF: Random forest
DT: Decision tree
AP: Anterior posterior
BMI: Body mass index
SE: Sensitivity
TP: True positive
SP: Specificity
TN: True negative
PR: Precision
ACC: Accuracy
FN: False negative
FP: False positive
MCCV: Monte Carlo cross validation
